# Ticagrelor-induced Angioedema After Percutaneous Coronary Intervention in a Patient with a History of Ischemic Stroke and Low Response to Clopidogrel: A Rare Dilemma

**DOI:** 10.7759/cureus.3720

**Published:** 2018-12-11

**Authors:** Paramarajan Piranavan, Nirmal J Kaur, Fady Marmoush, Andrew Burton, Joseph Hannan

**Affiliations:** 1 Internal Medicine, Saint Vincent Hospital, Worcester, USA; 2 Cardiology, Saint Vincent Hospital, Worcester, USA

**Keywords:** angioedema, ticagrelor, dual antiplatelet therapy

## Abstract

Dual antiplatelet therapy (DAPT) is widely recognized as the mainstay of treatment after percutaneous coronary intervention (PCI). Premature discontinuation may pose a risk of in-stent thrombosis, acute myocardial infarction, and death. With the increased usage of antiplatelet agents, increased attention has been drawn to their potential allergic reactions.

A 66-year-old male with a history of coronary artery disease and ischemic stroke was admitted with worsening severity angina for cardiac catheterization. He was on dual antiplatelet agents, clopidogrel, and aspirin prior to admission. He had PCI and a drug-eluting stent deployment to the culprit vessel. Due to low responsiveness to clopidogrel, he was started on ticagrelor, as prasugrel was contraindicated due to the history of ischemic stroke. A few hours after ticagrelor initiation, he developed shortness of breath, swelling of the throat and tongue, and was diagnosed with angioedema. He didn't have any prior reported history of allergy to any medications to the contrast medium or heparin. The offending medication, ticagrelor, was discontinued. He was managed with intravenous steroids and antihistamines. After the resolution of angioedema, he was discharged with double the dose of clopidogrel in addition to aspirin. The patient did not have any ischemic symptoms or coronary events for the following six-month period of follow-up.

The case highlights a relatively rare side effect of ticagrelor. Health care providers should be vigilant about the angioedema following ticagrelor administration. In our patient, it was effectively managed by discontinuing the offending medication and the administration of steroids and histamine blockers. The recovery was prompt, without any serious untoward effects. The DAPT was changed to clopidogrel, double the conventional dose, in addition to aspirin.

## Introduction

Dual antiplatelet therapy (DAPT) is widely recognized as the mainstay of treatment after percutaneous coronary intervention (PCI) [1]. Premature discontinuation may pose a risk of in-stent thrombosis, acute myocardial infarction, and death [2]. The addition of clopidogrel to aspirin monotherapy reduced the one-year incidence of cardiovascular events by 20% [3]. Ticagrelor and prasugrel have shown more potent inhibition of the P2Y12 receptors of platelets and have demonstrated improved efficacy in comparison to clopidogrel in the platelet inhibition and patient outcomes (PLATO) and therapeutic outcomes by optimizing platelet inhibition with prasugrel (TRITON) trials [4-5]. Furthermore, a low response to clopidogrel has been demonstrated to pose an independent risk for in-stent thrombosis [6-7]. Ticagrelor, which reversibly inhibits the adenosine diphosphate (ADP) P2Y12 receptor, has demonstrated improved outcomes, without an increased risk of bleeding, when compared to clopidogrel [5]. Allergic reactions to antiplatelet drugs are not uncommon. The reported prevalence of aspirin-exacerbated respiratory tract diseases is 10% and aspirin-induced urticarial rashes are reported in 0.07%-0.2% of cases [8]. With the increased usage of antiplatelet agents for the treatment of cardiovascular disease, increased attention has been drawn to their potential allergic reactions. Hypersensitivity reactions have been reported in 6% of patients with clopidogrel usage, 1.5% of whom require discontinuation [9]. To our knowledge, only six cases of ticagrelor hypersensitivity have been reported to date [10-15]. Reporting rare adverse drug reactions to pharmacovigilance databases enables health care providers to access such data when needed [16]. We herein report a case of a patient with a history of ischemic stroke and low clopidogrel responsiveness who underwent PCI with drug-eluting stent placement and subsequently developed angioedema after receiving the first dose of ticagrelor.

## Case presentation

A 66-year-old male with a past medical history significant for hypertension, hyperlipidemia, ischemic stroke, coronary artery disease, and asthma was admitted for cardiac catheterization for worsening angina. He had symptoms of crescendo angina with New York Heart Association (NYHA) class IV symptoms and was referred by his primary cardiologist for coronary angiography with the intent to pursue revascularization as warranted. He had established coronary artery disease in 2012, with pharmacological myocardial perfusion imaging demonstrating inferior infarct and peri-infarct ischemia with an overall preserved systolic function for which medical treatment was pursued. In 2014, he reportedly underwent coronary angiography, which demonstrated a chronic total occlusion of the right coronary artery with grade III collaterals to the right coronary system and moderate non-obstructive disease of the left circumflex artery. In the interim, he ceased tobacco smoking and received guideline-directed medical therapy. He was doing well with optimal medical therapy, which included aspirin and clopidogrel, until three weeks prior to this presentation when he noted the onset of recurrent angina. Angina initially occurred with mild exertion, subsequently progressed to angina at rest, and he was admitted to hospital with unstable crescendo angina.

**Figure 1:**
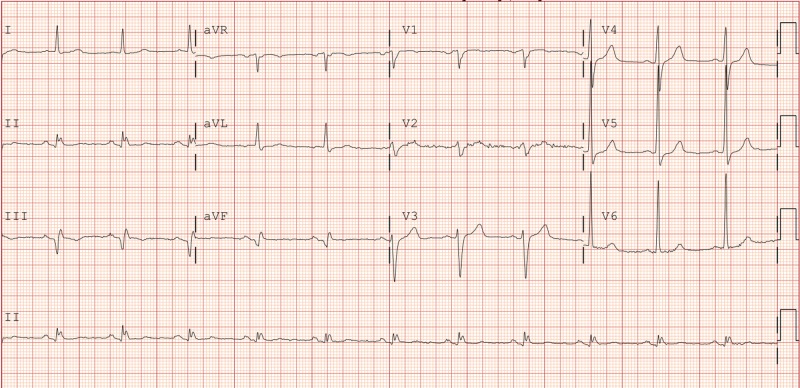
EKG on admission EKG: electrocardiogram

On admission, his vital signs were stable. His physical exam was unremarkable. A 12-lead electrocardiogram (EKG) demonstrated inferior Q waves and left ventricular hypertrophy without acute ST-T abnormalities (Figure 1). Cardiac biomarkers were normal (peak creatinine phosphokinase-MB (CK MB) fraction and troponin T were 9.9 ng/mL (normal range 0.0-10.4) and less than 0.03 ng/mL (<0.03 negative), respectively. Coronary angiography revealed multi-vessel coronary artery disease with a likely culprit lesion involving the proximal left circumflex artery (Videos 1-2). He underwent successful drug-eluting stent (DES) deployment to the proximal left circumflex artery (Video 3). PCI was uneventful and guideline-directed medical therapy was continued. Despite long-term adherence to clopidogrel, platelet reaction unit (PRU) was 235. Due to a high on treatment PRU, with levels greater than 208 associated with an increased risk of stent thrombosis [6-7], a decision was made to transition to ticagrelor. He received a single 180 mg loading dose of ticagrelor. Four hours after receiving ticagrelor, he complained of shortness of breath, throat pain, neck discomfort, and swelling of the tongue. His vital signs remained stable, and he did not exhibit any skin eruption. He demonstrated mild swelling of the tongue and significant swelling of his throat and uvula. No wheezing was noted on exam. He received supplemental oxygen and otolaryngology (ENT) consultation was sought. He was treated with dexamethasone, as well as H1 and H2 histamine blockers. After the first dose of treatment, his symptoms improved. He was closely monitored. The following morning, ENT performed flexible fiberoptic laryngoscopy, which revealed a mild edematous pharyngeal area. Based on the clinical scenario, a provisional diagnosis of ticagrelor-induced angioedema was entertained. He did not have any history of allergy to any medications, contrast medium, or heparin. The decision was made to discontinue ticagrelor. His symptoms improved over the next few days with a tapering dose of steroids and antihistamines. The final decision was made to double the dose of clopidogrel and the patient was discharged with 150 mg of clopidogrel and 81 mg of aspirin daily. He has not had any ischemic symptoms or coronary events over a six-month period of follow-up.

**Video 1:** Proximal stenosis in the left circumflex artery

**Video 2:** Post percutaneous coronary intervention of the left circumflex artery

**Video 3:** Percutaneous coronary intervention of the proximal left circumflex artery stenosis

## Discussion

Angioedema can be either hereditary or acquired and can be life-threatening when it involves the upper respiratory tract [17-18]. Immunologic, non-immunologic, and idiopathic subtypes of acquired angioedema have been described in the past [19]. Drug-induced angioedema is estimated to be less than 1% and is usually observed with angiotensin-converting enzyme inhibitors or angiotensin receptor blockers. Accumulation of bradykinin is implicated as the major contributor to the pathophysiology of angioedema [15]. Hypersensitivity reactions, in the form of rashes, to ticagrelor are rare (>1/1000, <1/100) and have been reported. The drug information portal (U.S National Library of Medicine) has a record of 34 cases, reported as side effects of rashes by the U.S Food and Drug Administration. Out of the two cases reported, angioedema usually occurred within 72 hours of exposure, and the same was the case with our patient [13,15]. Ticagrelor hypersensitivity can be either immediate type or delayed type. Desensitization attempts without fully establishing the underlying pathology can be detrimental [11].

With respect to the unique clinical scenario warranting DAPT for a protracted period, several therapeutic options can be debated. Our patient showed low responsiveness to clopidogrel and continuation of the same drug at the same dose would not be ideal. He developed angioedema four hours after the first dose of ticagrelor, indicating an immediate type of hypersensitivity. As we were unable to find any literature reports of possible ticagrelor desensitization protocols, and given his advanced age and despite insufficient data about the efficacy and safety of dual therapy with a higher daily dose of clopidogrel in the context of low responders to clopidogrel, we determined that an increased dose of clopidogrel remained a reasonable treatment choice.

Ticagrelor, prasugrel, and clopidogrel are ADP receptor (P2Y12) blockers, however, the chemical structures of these differ [16]. Ticagrelor is a direct, novel, reversible, non-competitive P2Y12 inhibitor [20]. Clopidogrel and prasugrel belong to the chemical group thienopyridines. Clopidogrel is turned into its active metabolite by the cytochrome P450 (CYP) system and its effectiveness depends on its metabolism. Efficacy in poor metabolizers is usually diminished. Prasugrel is a prodrug, and it is rapidly metabolized and effective in most individuals. However, in patients such as ours, with a history of ischemic stroke, prasugrel is contraindicated. The PLATO trial has shown ticagrelor is better than clopidogrel because it shows a significant reduction in death rate by principal causes and it has a comparatively lower bleeding risk [5]. A subgroup analysis suggested that elderly patients may derive greater benefit from treatment with ticagrelor than with clopidogrel [5].

Considering our dilemma of angioedema following treatment with ticagrelor, the presence of a contraindication to prasugrel due to a history of ischemic stroke and the demonstration of a low on treatment PRU following clopidogrel, we elected to continue clopidogrel at a higher dose of 150 mg daily. The patient tolerated the increased dose of clopidogrel without adverse ischemic or bleeding events at the six-month follow-up.

## Conclusions

In summary, we report a case of ticagrelor-induced angioedema occurring a few hours post drug initiation. This case highlights a relatively rare side-effect of ticagrelor. In our patient, it was effectively managed by discontinuing the offending medication and administrating steroids and histamine blockers. The recovery was prompt, without any serious untoward effects. An increased dose of clopidogrel was employed and well-tolerated.
